# Supplementary material for: Thrombotic Risk Detection in Patients with Polycythemia Vera: The Predictive Role of DNMT3A/TET2/ASXL1 Mutations
Source: Cancers (Basel). 2020 Apr 10;12(4):934. doi: 10.3390/cancers12040934 (PMC7226609; doi:10.3390/cancers12040934)
Supplement: Supplementary file 1 [file cancers-12-00934-s001.pdf]

cancers

Article

Thrombotic Risk Detection in Patients with Polycythemia Vera: the Predictive Role of *DNMT3A/TET2/ASXL1* Mutations

Supplementary Materials:

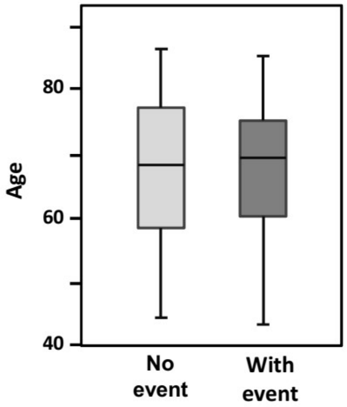

Group	*n*	Mean age	Median age	P25-P75	*p* value
No event	30	67.6	68.0	58.0-78.0	0.915
With event	25	67.2	69.0	59.5-75.5	

Figure S1. Box and whisker plot of age-matched PV patient case-control study cohort. There was no significant difference between the no event and with event groups of patients with PV in the case-control study. The band represents the median value; the whiskers represent the 25th (P25) and 75th percentiles (P75).

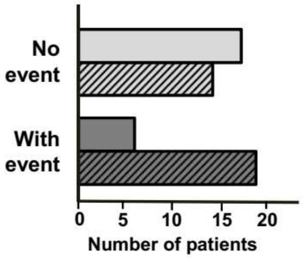

Group	*n*	No non-driver gene mutation (%)	Non-driver gene mutated (%)	*p* value
No event	30	17 (77.3)	13 (39.4)	0.0077
With event	25	5 (22.7)	20 (60.6)	
Total	55	22	23	

Figure S2. Frequency of non-driver gene mutations in the no thrombotic event and with thrombotic event groups of PV patients. Open bars represent no mutation in a non-driver gene, hatched bars represent non-driver gene mutated. Odds ratio 3.85 (95% confidence interval 1.207–12.251).

Table S1. Patient characteristics of whole MPN *n* = 68.

	Gender	Diagnosis MPN	Age at diagnosis	Driver gene mutation	Driver gene VAF	Occurrence of thrombosis before diagnosis	Event at diagnosis	Event post-diagnosis	DTA mutation	VAF DTA mutation	Other pathogenic non-driver mutations	VAF other non-driver mutation	Progression to AML	Exitus
1	F	MF	79	*JAK2*	53.2	no	no	no						
2	M	MF	75	TN					*ASXL1* c.1900_1922del p.E635Rfs*15	40.0	*SRSF2* c.284C>G p.P95R	41.2		
3	M	MF	82	TN		no	no	no			*U2AF1* c.101C>T p.S34F	48.1		25/04/2018
4	F	MF	32	*JAK2*	8.5	no	venous	no						
5	F	MF	69	*CALR*	39	yes	venous	no						
6	M	MF	75	*JAK2*	33.4	no	no	no						
7	M	MF	81	*JAK2*	40.7	no	no	no	*TET2* c.4546C>T p. R1516*	42.0	*SRSF2* c.284_307del p.P95_R102del	38.7		
8	F	MF	80	*MPL*	32.28	no	no	no	*TET2* c.4954C>T p.Q1652*	18.1				
9	M	MF	64	*JAK2*	15.8	yes	arterial	no						
10	M	MF	63	*JAK2*	70.8	no	no	no	*DNMT3A* c.1243C>T p.Q415*	46.7	*SF3B1* c.2098A>G p.K700E	43.4		23/07/2015
11	M	MF	48	*JAK2*	23.6	no	no	no						
12	M	MF	88	*CALR*	37.6	no	no	venous			*TP53* c.747G>C p.R249S	52.6		05/10/2016
13	F	MF	69	*JAK2*	30.8	no	no	no			*TP53* c.659A>G p.Y220C	59.2		
14	F	MF	75	*CALR*	31.4				*TET2* c.4317dupA p.R1440Tfs*38	3.2				
15	M	MF	44	*CALR*	36.5	no	no	no	*TET2* c.5650delA p.T1884Pfs*3	5.5				yes

	Gender	Diagnosis MPN	Age at diagnosis	Driver gene mutation	Driver gene VAF	Occurrence of thrombosis before diagnosis	Event at diagnosis	Event post-diagnosis	DTA mutation	VAF DTA mutation	Other pathogenic non-driver mutations	VAF other non-driver mutation	Progression to AML	Exitus
16	M	MF	70	*JAK2*	37.1	no	no	no	*ASXL1* c.2444_2447delTGTCinsCG p.L815Pfs*6/c.4334_4335delCT p.S1445*fs*1/c.2077C>T p.R693*	23.8 /3.2 /1.7	*U2AF1*c.101C>T p.S34F			
17	M	SMF-post PV	75	*JAK2*	55.5	no	no	no						
18	F	SMF-post PV	75	*JAK2*	82.2	no	venous	venous	*TET2* c.1683_1689delACCAGGA p.K561Nfs*5	38.3				
19	M	SMF-post PV	76	*JAK2*	15.9	no	no	no	*TET2* c.3594+1G>T p.?	13.4	*SRSF2* c.161C>A p.S54Y	11.1		
20	F	SMF-post PV	68	*JAK2*	57	no	no	no	*TET2*c.3500+1G>T p.?	18.9				
21	M	SMF-post ET		*CALR*	38.7	no	no	no			*NRAS* c.35G>A p.G12D	43.9		
22	F	SMF-post ET	49	*JAK2*	8.3	no	no	no						
23	M	SMF-post PV	84	*JAK2*	30.3	no	no	no						
24	M	SMF-post PV	42	*JAK2*	93.8	no	no	no			*NRAS* c.68T>C p.L23P	7.7		
25	F	SMF-post ET	88	*JAK2*	13.3	no	no	no			*RUNX1* c.883_884delTC p.S295Nfs*?, *SF3B1* c.1997A>C p.K666T	4.8, 40.7		

	Gender	Diagnosis MPN	Age at diagnosis	Driver gene mutation	Driver gene VAF	Occurrence of thrombosis before diagnosis	Event at diagnosis	Event post-diagnosis	DTA mutation	VAF DTA mutation	Other pathogenic non-driver mutations	VAF other non-driver mutation	Progression to AML	Exitus
26	M	SMF-post ET	54	*JAK2/CALR*	21.6	no	no	no						
27	F	SMF-post ET	68	*MPL*	66.2	no	no	no	*DNMT3A* c.1095C>A p.Y365*	42.3	*ETV6* c.577dupT p.S193Ffs*3	25.6		
28	F	ET	62	*JAK2/CALR*	10.9	no	no	no						
29	F	ET	37	TN		no	no	no						
30	F	ET	55	*MPL*	14.27	no	no	no						
31	F	ET	19	*MPL*	1.84	no	no	no						
32	F	ET	47	*JAK2*	19.2	no	venous	no						
33	F	ET	52	*JAK2*	2.6	no	venous	no						
34	F	ET	76	*JAK2*	40.3	yes	no	venous						
35	F	ET	37	*CALR*	30.2	no	venous	no						
36	F	ET	82	*CALR*	37.7	yes	no	arterial						
37	F	ET	66	*JAK2*	14	no	venous	no						
38	F	ET	68	*JAK2*	100	no	no	venous			*RUNX1* c.302T>G p.V101G	14.3		
39	F	ET	63	*MPL*	3.8	no	no	no						
40	F	ET	52	*JAK2*	23.6	no	no	venous						
41	F	ET	23	TN		no	no	no						
42	F	ET	40	TN		no	no	no						
43	M	ET	20	TN		no	no	no						
44	M	ET	56	TN		no	no	no	*ASXL1* c.1773C>A p.Y591*, *TET2* c.3202delC p.Q1068Kfs*14	48.9, 92.2				
45	F	ET	76	*JAK2*	27.9	no	no	no						19/02/2016
46	F	ET	73	TN		no	no	no	*DNMT3A* c.2583C>A p.C861*	30.5				yes
47	M	ET	79	*CALR*	12.6	no	no	no						

	Gender	Diagnosis MPN	Age at diagnosis	Driver gene mutation	Driver gene VAF	Occurrence of thrombosis before diagnosis	Event at diagnosis	Event post-diagnosis	DTA mutation	VAF DTA mutation	Other pathogenic non-driver mutations	VAF other non-driver mutation	Progression to AML	Exitus
48	F	ET	68	*CALR*	28.1		no	no						
49	M	ET	72	*JAK2*	30.7	yes	no	venous, arterial	*DNMT3A* c.1430-2A>C p.?	44.8	*CEBPA* c.355_356delGT p.V119Hfs*50	7.5		
50	M	ET	36	*JAK2*	3.7	no	arterial	no						
51	F	ET	27	*JAK2*	24.7	no	no	no						
52	F	ET	46	*JAK2*	13.7	no	no	no						
53	M	PV	47			no	no	no						
54	M	PV	53			no	no	no						
55	F	PV	73	*JAK2*	61.8	no	no	no	*DNMT3A* c.2645G>A p.R882H	46.7	*TP53* c.524G>A p.R175H /c.722C>T p.S241F	19.9 /64.8		
56	F	PV	70	*JAK2*	27.9	yes	arterial	no	*DNMT3A* c.958C>T p.R320*	50.3				
57	F	PV	74	*JAK2*	36.7	no	no	no					yes	21/12/2019
58	M	PV	38	*JAK2*	30.7	no	no	venous	*ASXL1* c.4015G>T p.G1339*, *TET2* c.5458delA p.S1820Vfs*13	1.2, 3.2				
59	F	PV	73	*JAK2*	74.4	no	no	no						
60	M	PV	61	*JAK2*	49.9	yes	arterial	no	*DNMT3A* c.73-227_177+479del splice_donor_cds_indel	1.3				
61	F	PV	86	*JAK2*	49.9	yes	no	no	*TET2* c.1852C>T p.Q618*/c.1630C>T p.R544*	48.0 /45.8				
62	M	PV	60	*JAK2*	23.8	yes	no	no	*ASXL1* c.1900_1922del p.E635Rfs*15	35.3				09/12/2016

	Gender	Diagnos is MPN	Age at diagnosis	Driver gene mutation	Drive r gene VAF	Occurrence of thrombosis before diagnosis	Event at diagnosis	Event post-diagnosis	DTA mutation	VAF DTA mutation	Other pathogenic non-driver mutations	VAF other non-driver mutation	Progression to AML	Exitus
63	F	PV	73	*JAK2*	47.7	yes	venous	no	*TET2* c.3782G>C p.R1261P	28.1				09/12/2016
64	F	PV	80	*JAK2*	34.6	no	no	no	*TET2* c.5650A>G p.T1884A	42.9				
65	F	PV	64	*JAK2*	60.4	no	no	venous	*TET2* c.5354delA p.K1785Rfs*35	2.0				
66	F	PV	83	*JAK2*	77.2	no	no	no			*RUNX1* c.1265_1266del AG p.E422Afs*?	28.9		yes
67	M	PV	68	*JAK2*	12.9	no	no	no	*ASXL1* c.1772dupA p.Y591*fs*1	5.2				
68	M	PV	90	*JAK2*	86.7	no	no	no			*SRSF2* c.284C>T p.P95L	44.1		

F: female, M: male, TN: triple negative, VAF: variant allele frequency, DTA: *DNMT3A*/*TET2*/*ASXL1*, AML: acute myeloid leukemia

Table S2. Frequency of mutation in 1 of the 30 genes included in the myeloid panel (including the driver genes *JAK2, CALR* and *MPL*) for the whole MPN patient series.

MPN	N patients	At least 1 mutation % (*n*)	More than 1 mutation % (*n*)	% patients with mutation (*n*)	Average VAF %
PV	16	75.0 (12)	25 (4)	25 (4)	28
PMF	16	62.5 (10)	25 (4)	37.5 (6)	34.5
SMF	11	72.7 (8)	36.4 (4)	27.3 (3)	43.9
ET	25	16 (4)	8 (2)	84 (21)	57.4
Total	68	50 (34)	19.1 (13)	50 (34)	41.0

ET: essential thrombocythemia; MPN: myeloproliferative neoplasm; PMF: primary myelofibrosis; PV: polycythemia vera; SMF: secondary myelofibrosis; VAF: variant allele frequency.

Table S3. Contingency table for Pearson's χ2 test to evaluate the association between *JAK2* mutation and thrombotic event in patients with ET, PMF and SMF post-ET ($n = 44$).

	No event (%)	Event (%)	Total	*p* value
***JAK2* WT**	16 (53.3)	4 (28.6)	20	0.124
***JAK2* mutated**	14 (46.7)	10 (71.4)	24	
Total	30	14	44	

ET: essential thrombocythemia; PMF: primary myelofibrosis; SMF: secondary myelofibrosis; WT: wild type.

Table S4. Contingency tables for Pearson's χ2 test to evaluate the association between thrombotic event and DTA mutation or any non-driver mutation in *JAK2*-mutated versus *JAK2* wild-type patients with ET, PMF and SMF post-ET ($n = 44$).

	***JAK2* mutated**				***JAK2* WT**			
	**No event (%)**	**Event (%)**	**Total**	***p* value**	**No event (%)**	**Event (%)**	**Total**	***p* value**
**DTA WT**	11 (78.6)	9 (90)	20		11 (68.8)	4 (100)	15	
**DTA mutated**	3 (21.4)	1 (10)	4	0.459	5 (31.3)	0 (0)	5	0.197
Total	14	10	24		16	4	20	
**Non-driver WT**	9 (64.3)	8 (80)	17		8 (50)	3 (75)	11	0.369
**Non-driver mutated**	5 (35.7)	2 (20)	7	0.404	8 (50)	1 (25)	9	
Total	14	10	24		16	4	20	

DTA: *DNMT3A*/*TET2*/*ASXL1;* ET: essential thrombocythemia; PMF: primary myelofibrosis; SMF: secondary myelofibrosis; WT: wild type.

Table S5. Patient characteristics of age-matched PV case-control cohort, *n* = 55.

	Gender	Diagnosis	Age at diagnosis	Occurrence of thrombosis before diagnosis	Event pre-diagnosis	Event post-diagnosis	*JAK2* VAF	DTA mutation	VAF DTA mutation	Other pathogenic non-driver mutations	VAF other pathogenic non-driver mutations	Progression to AML	Exitus
1	F	PV	73	no	no	no	61.83	*DNMT3A* c.2645G>A p.R882Hs	46.73	*TP53* c.524G>A p.R175H/c.722C>T p.S241F	19.88/ 64.79		
2	F	PV	70	yes	arterial	no	27.9	*DNMT3A* c.958C>T p.R320*	50.3				
3	F	PV	74	no	no	no	36.7					yes	21/12/2019
4	M	PV	38	no	no	venous	30.7	*ASXL1* c.4015G>T p.G1339*, *TET2* c.5458delA p.S1820Vfs*13	1.2, 3.2				
5	F	PV	73	no	no	no	74.4						
6	M	PV	61	yes	arterial	no	49.9	*DNMT3A c.73-*227_177+479del splice_donor_cds_indel	1.3				
7	F	PV	86	yes	no	arterial	49.9	*TET2* c.1852C>T p.Q618*/c.1630C>T p.R544*	48/ 45.8				
8	F	PV	73	yes	venous	no	47.7	*TET2* c.3782G>C p.R1261P	28.1				09/12/2016
9	F	PV	80	no	no	no	34.6	*TET2* c.5650A>G p.T1884A	42.9				
10	F	PV	64	no	no	venous	60.4	*TET2* c.5354delA p.K1785Rfs*35	2.0				
11	F	PV	83	no	no	no	77.2			*RUNX1* c.1265_1266delAG p.E422Afs*?	28.9		
12	F	SMF-post PV	61	no	venous	venous	82.2	*TET2* c.1683_1689delACCAGGA p.K561Nfs*5	38.3				

	Gender	Diagnosis	Age at diagnosis	Occurrence of thrombosis before diagnosis	Event pre-diagnosis	Event post-diagnosis	*JAK2* VAF	DTA mutation	VAF DTA mutation	Other pathogenic non-driver mutations	VAF other pathogenic non-driver mutations	Progression to AML	Exitus
13	M	SMF-post PV	65	no	no	no	15.9	*TET2* c.3594+1G>T p.?	13.4	*SRSF2* c.161C>A p.S54Y	11.1		
14	F	SMF-post PV	59	no	no	no	57	*TET2* c.3500+1G>T p.?	18.9				28/05/2019
15	M	SMF-post PV	62	no	no	no	30.3	*DNMT3A* c.2255T>A p.F752Y, *TET2* c.3578G>A p.C1193Y	1.3, 1.0				
16	F	SMF-post PV	42	no	no	no	93.8			*NRAS* c.68T>C p.L23P	7.7		13/05/2018
17	M	PV	62	no	no	venous	12.9	*TET2* c.1630C>T p.R544*	5.2				
18	M	PV	44	no	no	venous	96.5	*TET2* c.3260delC p.S1087Ffs*19	47.6				
19	F	PV	47	no	no	no	16.8						
20	F	PV	59	yes	venous	venous, arterial	40.5	*TET2* c.2746C>T p.Q916*/c.4315A>T p.K1439*/c.3845G>A p.G1282D	40.1/ 9.8/ 18.9				
21	M	PV	69	no	no	no	34.6	*TET2* c.3508C>T p.Q1170*/ c.3660delC p.C1221Vfs*5/c.1971dupA p.H658Tfs*23	12.3/ /1.8 /1.8				21/04/2016
22	F	PV	79	no	no	no	14.6			*TP53* c.783-2A>T p.?	4.0		
23	F	PV	86	no	no	no	50.4	*TET2* c.1815_1817delCACinsAA p.Y605*fs*1	6.8				
24	M	PV	83	no	no	no	38.8	*ASXL1* c.1636C>T p.Q546*, *TET2* c.4119_4123delATGTT p.C1374Gfs*25	42.4, 37.6	*SRSF2* c.284C>A p.P95H	43.1		

	Gender	Diagnosis	Age at diagnosis	Occurrence of thrombosis before diagnosis	Event pre-diagnosis	Event post-diagnosis	*JAK2* VAF	DTA mutation	VAF DTA mutation	Other pathogenic non-driver mutations	VAF other pathogenic non-driver mutations	Progression to AML	Exitus
25	F	PV	78	no	no	no	17.3	*DNMT3A* c.1701_1704delTCGC p.K569Afs*57, *TET2* c.3847_3880del p.A1283Tfs*69	44.2, 36.8				
26	M	PV	45	no	no	no	17						
27	F	PV	58	no	no	no	84.1						
28	F	PV	66	yes	arterial	no	11.9	*ASXL1* c.2077C>T p.R693*	23.3				
29	M	PV	63	yes	arterial	no	63.7	*DNMT3A* c.1211+1G>A p.?/ c.1395+1G>T p.?	32.9/ 2.3				
30	M	PV	83	yes	venous, arterial	no	5.4	*DNMT3A* c.2189G>A p.R730H/ c.2255C>Tp.P752L/ c.1367dupTp.F457Lfs*3	16.7/ 3.1/ 1.5				
31	M	PV	42	yes	arterial	no	22.2						
32	M	PV	66	no	no	no	72.8						
33	M	PV	54	no	no	no	31.5						
34	M	PV	68	yes	arterial	venous, arterial	75	*TET2* c.3333_3334del p.L1111Ffs*18	44.7				
35	F	PV	59	no	no	no	20.8, 18.2					yes	
36	F	PV	50	no	no	venous, arterial	94.7						04/03/2011
37	M	PV	63	no	no	no	52.9						29/10/2015
38	F	PV	67	no	no	no	38.3						30/04/2008
39	F	PV	74	yes	no	venous	58.2	*DNMT3A* c.1270del p.I424Lfs*75, *TET2* c.346C>Tp.Q116*/ c.1482del p.T495Qfs*2	46.3, 10.5/ 4.3				
40	M	PV	53	yes	no	arterial	38.1, 63.2			*EZH2* c.2188C>T p.Q730*	13.4		06/01/2014

	Gender	Diagnosis	Age at diagnosis	Occurrence of thrombosis before diagnosis	Event pre-diagnosis	Event post-diagnosis	*JAK2* VAF	DTA mutation	VAF DTA mutation	Other pathogenic non-driver mutations	VAF other pathogenic non-driver mutations	Progression to AML	Exitus
41	M	PV	74	no	no	no	17.2						29/08/2017
42	M	PV	50	yes	no	venous	55.3						13/11/2017
43	F	PV	49	no	no	no	74.1			*TP53* c.80C>T p.P27L	1.7		
44	F	PV	75	no	no	no	49.3, 2.5						29/04/2014
45	M	PV	66	no	arterial	no	23.8	*ASXL1* c.2771_2781delinsT p. P924Lfs*18)	3.3	*SF3B1* c.1998G>T p.K666N	40.9		
46	F	PV	80	yes	arterial	no	33.8, 47.2	*DNMT3A* c.1484T>A p.L647H	6.9				
47	M	PV	56	no	no	no	82.5						
48	M	PV	55	no	no	no	75.7						05/10/2018
49	F	PV	54	no	no	no	44.3	*DNMT3A* c.2598-3_2599del splice_donor_cds_indel, *TET2* c.3764dupA p.Y1255*	4.6, 1.4				
50	F	PV	67	yes	arterial	arterial	32.8	*TET2* c.2926C>T p.Q976*	3.5	*EZH2* c.1680_1681insA p.R561Tfs*26, *TP53* c.831T>G p.C277W/ c.427G>A p.V143M	1.3, 3.5/ 1.2	yes	
51	F	PV	72	no	no	no	66.8, 31.1					yes	
52	F	PV	66	yes	no	arterial	24.9	*TET2* c.1876C>T p.Q626*	22.4				
53	M	PV	55	yes	arterial	no	62.7						

	**Gender**	**Diagnosis**	**Age at diagnosis**	**Occurrence of thrombosis before diagnosis**	**Event pre-diagnosis**	**Event post-diagnosis**	***JAK2* VAF**	**DTA mutation**	**VAF DTA mutation**	**Other pathogenic non-driver mutations**	**VAF other pathogenic non-driver mutations**	**Progression to AML**	**Exitus**
54	F	PV	83	no	no	no	60.1	*DNMT3A* c.1700_1714del p.V567_A571del, *TET2* c.3782G>A p.R1261H	44.1, 43.6	*SRSF2* c.284C>A p.P95H	2.3		
55	F	PV	69	yes	arterial	no							

F: female, M: male, TN: triple negative, VAF: variant allele frequency, DTA: *DNMT3A*/*TET2*/*ASXL1*, AML: acute myeloid leukemia
